# A genetic assessment of the English bulldog

**DOI:** 10.1186/s40575-016-0036-y

**Published:** 2016-07-29

**Authors:** Niels C. Pedersen, Ashley S. Pooch, Hongwei Liu

**Affiliations:** 1 Center for Companion Animal Health, School of Veterinary Medicine, University of California, One Shields Avenue, Davis, CA 95616 USA; 2 School of Veterinary Medicine, University of California, One Shields Avenue, Davis, CA 95616 USA

**Keywords:** English bulldog, Health, Genetic assessment, Short tandem repeats (STRs), Maternal haplotypes, Paternal haplotypes, Internal relatedness (IR), Dog leukocyte antigen (DLA), DLA class I and II, Runs of homozygosity (ROH)

## Abstract

**Background:**

This study examines genetic diversity among 102 registered English Bulldogs used for breeding based on maternal and paternal haplotypes, allele frequencies in 33 highly polymorphic short tandem repeat (STR) loci on 25 chromosomes, STR-linked dog leukocyte antigen (DLA) class I and II haplotypes, and the number and size of genome-wide runs of homozygosity (ROH) determined from high density SNP arrays. The objective was to assess whether the breed retains enough genetic diversity to correct the genotypic and phenotypic abnormalities associated with poor health, to allow for the elimination of deleterious recessive mutations, or to make further phenotypic changes in body structure or coat. An additional 37 English bulldogs presented to the UC Davis Veterinary Clinical Services for health problems were also genetically compared with the 102 registered dogs based on the perception that sickly English bulldogs are products of commercial breeders or puppy-mills and genetically different and inferior.

**Results:**

Four paternal haplotypes, with one occurring in 93 % of dogs, were identified using six Y-short tandem repeat (STR) markers. Three major and two minor matrilines were identified by mitochondrial D-loop sequencing. Heterozygosity was determined from allele frequencies at genomic loci; the average number of alleles per locus was 6.45, with only 2.7 accounting for a majority of the diversity. However, observed and expected heterozygosity values were nearly identical, indicating that the population as a whole was in Hardy-Weinberg equilibrium (HWE). However, internal relatedness (IR) and adjusted IR (IRVD) values demonstrated that a number of individuals were the offspring of parents that were either more inbred or outbred than the population as a whole. The diversity of DLA class I and II haplotypes was low, with only 11 identified DLA class I and nine class II haplotypes. Forty one percent of the breed shared a single DLA class I and 62 % a single class II haplotype. Nineteen percent of the dogs were homozygous for the dominant DLA class I haplotype and 42 % for the dominant DLA class II haplotype. The extensive loss of genetic diversity is most likely the result of a small founder population and artificial genetic bottlenecks occurring in the past. The prominent phenotypic changes characteristic of the breed have also resulted in numerous large runs of homozygosity (ROH) throughout the genome compared to Standard Poodles, which were phenotypically more similar to indigenous-type dogs.

**Conclusions:**

English bulldogs have very low genetic diversity resulting from a small founder population and artificial genetic bottlenecks. Although some phenotypic and genotypic diversity still exists within the breed, whether it is sufficient to use reverse selection to improve health, select against simple recessive deleterious traits, and/or to accommodate further genotypic/phenotypic manipulations without further decreasing existing genetic diversity is questionable.

## Plain English Summary

The English bulldog is one of the most popular breeds in the world because of its child-like appearance and demeanor. The alterations in body type and behavior needed to create the breed have required physical changes well beyond its village dog ancestors. These changes have occurred over hundreds of years but have become particularly rapid over the last decades. Unfortunately, popularity does not equate to health and there have been increasing pressures on breeders to moderate the extreme physical changes that now affect the breed and its health. Improving health through genetic manipulations presumes that enough diversity still exists to improve the breed from within, and if not, to add diversity by outcrossing to other breeds. The present study was an assessment of genetic diversity that still exists in a representative number of individual English bulldogs using DNA rather than pedigrees. The results confirm that the breed has lost considerable genetic diversity through such things as small founder population and artificial genetic bottlenecks resulting from highly focused selection for specific desired physical traits. This is manifested by a narrowing of allele diversity in many parts of the genome, and the creation of numerous large regions of the genome that are essentially identical within the breed, which are significantly different from other dogs. Loss of genetic diversity is also pronounced in the region of the genome that contains many of the genes that regulate normal immune responses. The loss of genetic diversity and extreme changes in various regions of the genome will make it very difficult to improve breed health from within the existing gene pool. Loss of present genetic diversity is further threatened by rapid integration of new coat color mutations, increased wrinkling of the coat, and attempts to create a more compact body type. Contrary to current beliefs, brachycephaly and the resulting breathing problems in the breed are the result of complex changes in head structure, and cannot be corrected by merely lengthening the face. Furthermore, other issues in English bulldogs need to be addressed, including many serious health problems that are not associated with brachycephaly, but are intrinsic to inbreeding.

## Background

The first mention of what might be a contemporary type bulldog was reported in 1632 [1]. The “bull” refers to the breed’s use in the sport of bull baiting in England. Bull baiting, which had its beginning even centuries earlier with the Egyptians, Greeks and Romans, became a national sport in England from the 13^th^ to 18^th^ centuries [1]. The objective was for the dog to latch onto the bull’s nose and force it to the ground, with the first dog to do so the victor. These original bulldogs had stockier bodies, larger heads and stronger jaws, and a more ferocious and aggressive temperament than the common indigenous dogs of the period. Therefore, their ancestors were presumably mastiff-type dogs originally bred in Asia for their strength and aggressiveness. Controversy exists as to whether these Mastiffs were crossed with breeds such as the Pug to make them more effective at bull baiting [1]. The first description of a Bulldog as a distinct entity from the Mastiff was in 1631 in a letter written from Mr. Eaton in Spain to a friend in England [1]. The bulldog was further genetically altered over the 500 year period of bull baiting by “selection of the fittest,” with emphasis on increasing agility and putting more power and weight into the head and front end to minimize spinal damage when they were shaken by the bulls [1].

Attempts to legislate against bull baiting began in the UK in 1802 and the sport was finally abolished by an Act of Parliament in 1835, which led the breed to the brink of extinction [1]. The Bulldog endured by the efforts of a small group of aficionados and the breed underwent even more change in size and temperament after 1835 to ultimately make them into the shorter-faced, squatter and more affable companion dog that we know today.^1^ The first Bulldogs appeared in show rings in the UK in 1860, and the Bulldog was first recognized by the American Kennel Club in 1886.^2^

A number of modern breeds use “bull” or “bulldog” in their names and all have evolved from the original Bulldogs and Mastiff-type dogs [1, 2]. Some of these breeds are of more ancient origin, while others are reconstructions of breeds that no longer exist. The modern Olde English Bulldogge is a reconstruction of the original bulldog based on crosses between English bulldog, American bulldog, American Pit Bull Terrier and Mastiff.^3^ The Miniature bulldogs, French bulldog and American bulldog are also constructed breeds that nonetheless trace some of their ancestry to the original English bulldog. Although there are several “bulldogs,” the ideal English bulldog is easily differentiated by its huge head with wedge-shaped body, short and folded ears; stocky build with deep furrows of the skin, especially of the face; short or corkscrew tail; short thick legs with equally broad paws; and a gentle, child-like appearance and disposition [2].

**Fig. 1:**
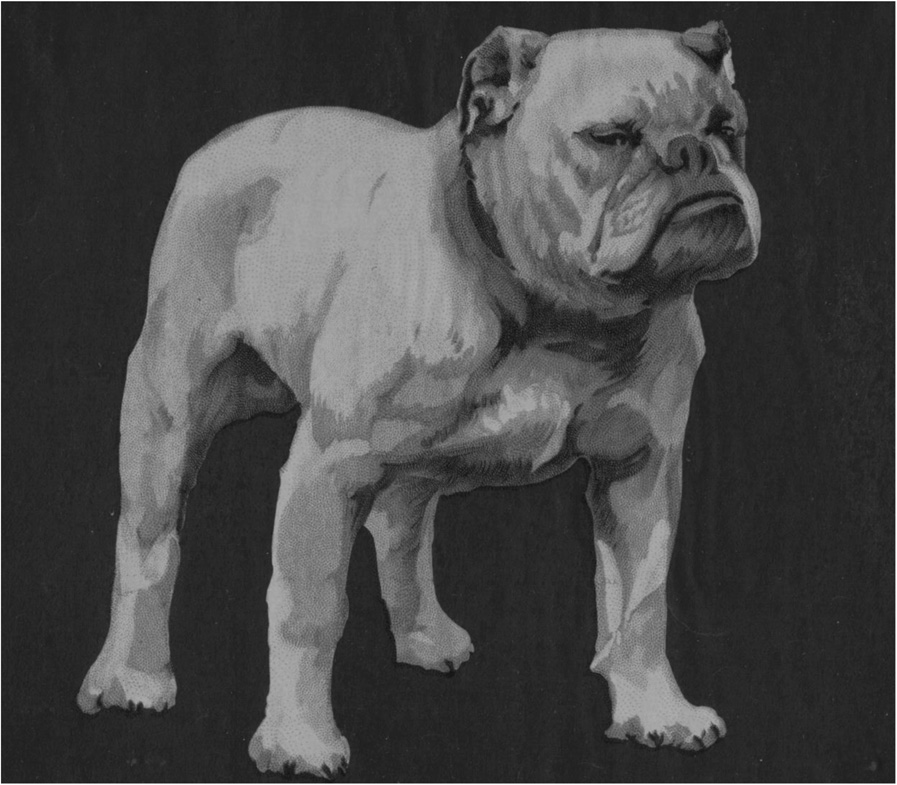
A drawing of an English bulldog from 1935 copied from a California Orange Crate Label of the Rudolph Marketing Company.

The outward appearance of many dog breeds change with time, and this is also true of the English bulldog.^4^^,^^5^ Photographs of English bulldogs from the nineteenth century depict dogs with less pronounced brachycephaly, less chondrodystrophic skeletal structure, a long tail, and without excessive skin folds on their face or body.^6^ A picture of an English bulldog from 1935 can be found on an orange crate label advertising California oranges (Fig. 1) and this dog was already differing in appearance from its ancestors pictured a century earlier and from contemporary English bulldogs. However, photographs of modern English bulldogs still demonstrate a range of phenotypic diversity, with some dogs being smaller and squatter or larger and longer-legged, some with smooth rather than wrinkled coats, some with less brachycephalic and furrowed faces, some with longer and less chondrodystrophic limbs, some more prognathous than others, some with intact tails, and others with different shaped remnants of tails.^7^^,^^8^

Although much has been said about the ill-health of contemporary English Bulldogs, the English bulldog of the late 19^th^ century also had its share of health problems and critics.^5^ Therefore, the English bulldog was not initially popular in the UK, although its popularity has greatly increased over the last decades in many affluent countries [3]. English bulldog owners love their baby-like appearance and demeanor and the breed has been cited as one of the prime examples of exuberant anthropomorphism [4]. Health problems of the breed have not decreased its popularity and deviations from the original standard based on changing perceptions of “champion form” have led to even more conformational changes that have further affected breed health.^5^

The health problems of the English bulldog have been well documented and start with conception, fetal development and parturition. Severe conformational changes have necessitated a high rate of artificial insemination and Caesarean sections and litter sizes tend to be small.^5,^^9^ The breed ranks second in congenital disease and associated puppy mortality [5], due mainly to birth defects such as flat chests with splayed legs; anasarca (water babies) and cleft palate [6].^10^^,^^11^ Although some English bulldogs enjoy reasonable health, their longevity is definitely affected by the degree of conformational change and inbreeding, which is reflected by lifespan estimates ranging from 3.2 to 11.3 years with a median of 8.4 years [7]. Individuals requiring extensive veterinary care at a young age rarely live beyond 5–6 years of age, leading to a bimodal mortality curve for the breed.^11^

The brachycephalic syndrome is a leading cause of ill-health and death in the breed. However, the syndrome is not caused by brachycephaly per se, as brachycephalic breeds such as the boxer do not suffer the syndrome to the same degree. The bulldog tongue is excessively large at the base, the palate is large and easily obstructed by the base of the tongue, the lower jaw is pushed forward (prognathous), and the nares are frequently stenotic and the trachea hypoplastic. This leads to loud panting during physical exercise, stridor during rest and slobbering; sleep apnea, hypercapnia and hypochloremia/hypomagnesemia; exercise intolerance, cyanosis and collapse; and choking fits manifested by gagging, retching, vomiting, aerophagia/flatulence and aspiration pneumonia [8–12]. The breathing difficulties of English bulldogs also make them very sensitive to overheating and heat stroke.

Chondrodysplasia, a heritable skeletal disorder that has been incorporated into the phenotype of many dog breeds [13], predisposes English bulldogs to skeletal disorders such as hip dysplasia, elbow dysplasia, luxating patella and shoulders, intervertebral disk disease, cruciate ligament rupture, hemivertebra, torsional pelvic deformity and problems with normal copulation and parturition [14, 15]. Prognathism predisposes to dental disease, while excessive folding of the skin, especially on the face, is associated with skin fold dermatitis, muzzle acne, folliculitis, furunculosis, and eye conditions such as entropion, ectropion, and eversion of the third eyelid. The cork-screw tail can result in tail fold dermatitis. Other heritable conditions that are related to loss of genetic diversity and inbreeding include cataract, various heart valve defects including pulmonic stenosis, hydrocephalus, cysteine urolithiasis, and hiatal hernias; immunologic disorders that include a propensity for severe demodectic mange indicative of immunodeficiency, allergies associated with atopic dermatitis and ear infections, and autoimmune diseases such as hypothyroidism; and cancers including glioblastoma, mast cell sarcoma and lymphoma [15, 16]. Although the bond and affection between English bulldogs and their owners is strong, the cost of treating health problems is often prohibitive and many of them end up in shelters or euthanized.

Severe health problems in English bulldogs in the USA and the UK have prompted articles [16] such as “Can the Bulldog be saved?” The article documented the short lives of University of Georgia mascots and re-kindled the humane issues of knowingly breeding dogs destined to lives of serious ill-health. An earlier broadcast on the BBC spawned three independent reports identifying the English bulldog as a breed in need of genetic intervention, presumably to breed away from the extremes of brachycephaly and chondrodysplasia and increase genetic diversity. The British Kennel Club responded to these criticisms by revising the English bulldog standard to select against obvious breathing difficulties and avoid extreme facial wrinkling.^12^ However, bulldog breeders in the UK argue that “what you’ll get is a completely different dog, not a British bulldog”^12^, a strange argument given the continuous phenotypic changes that have occurred within the breed over many decades.^6,7,8^ Groups in The Netherlands have called for a ban on English bulldogs based on a belief that the breed can no longer be returned to a health, while supporters believe that the breed can be returned to health from within and are petitioning the government against the ban.^13^ American breeders of English bulldogs have avoided the issue and even deny that the breed is unhealthy, pointing to its popularity as proof [3]. However, the bulldog has been banned from plane travel in the cargo hold by many domestic and international airlines due to a high incidence of deaths.^14^

English bulldogs have risen from 5^th^ to 4^th^ in popularity between 2013 and 2015 in AKC registrations [3], proving that the public is more enamored with the dog than concerned about its health. Assuming that this attitude will change and steps taken to improve the breed’s health, how can this best be accomplished? Diehard breeders would argue that this should involve genetic change from within existing bloodlines. The question then becomes whether there is enough residual phenotypic and genotypic diversity to significantly improve the health of the breed using the existing stock. Although there is still phenotypic variability in the breed based on photographs^6,7,8^, bulldogs that reproduce without assistance, move freely, walk or run for long distances, and breathe normally even at rest are the exception.

Efforts are underway in the UK to improve the health of bulldogs from within the breed by making modest changes on what is acceptable in a show dog. However, there is very little knowledge about the actual genetic status of the breed and whether enough genetic and phenotypic diversity still exists to significantly improve health without further reducing existing genetic diversity or relying on outcrossing. Breeders still rely heavily on pedigrees and coefficients of inbreeding (COI). The problem is that pedigrees emphasize ancestors and inheritance by descent but not actual genetic makeup. They are also subject to parentage errors and COIs based on a few recent generations are of limited value in a breed that started with few founders subjected to numerous artificial bottlenecks that occurred decades and even centuries earlier. Therefore, we endeavored to conduct a broad-based genetic assessment of the breed using DNA rather than pedigrees. The findings of this study indicate that English bulldogs may have insufficient genetic diversity to significantly improve gross physical abnormalities associated with poor health, to eliminate simple recessive deleterious traits, or to use inbreeding to rapidly integrate new coat colors and to breed for a smaller and more compact shape without further decreasing genetic diversity in individuals and adding to their health problems.

## Methods

### Sample acquisition and DNA extraction

The Veterinary Genetics Laboratory (VGL) (UC Davis School of Veterinary Medicine, Davis, CA, USA) provided DNA samples of 102 registered English Bulldogs; 87 of the dogs were from the USA, six from Finland, three each from Canada and Austria, and one each from Czechoslovakia, Hungary, and Argentina. DNA from these dogs was submitted mainly for coat color or hyperuricosuria mutation testing and used in breeding programs. As such, they were presumed to be of adequate health and therefore listed as “controls”. Thirty-seven DNA samples were collected from whole blood of English bulldogs submitted for various diagnostic tests at the UC Davis Veterinary Medical Teaching Hospital (VMTH). These dogs were seen for a variety of health problems ranging from breathing problems, eye problems, skin disorders, orthopedic problems, or cancer and were therefore listed as “case”. DNA was extracted using established procedures [17].

### Determination of maternal and paternal haplotypes

Maternal haplotypes were determined by sequencing 655 bp of the mitochondrial D-loop (nucleotide 15453–16107) in 48 English Bulldogs as described [18]. Dogs were as unrelated as possible based on genomic STR markers. Sequences were analyzed using Geneious software [19]. Final sequences were compared to the National Center for Biotechnology Information (NCBI) database using nucleotide Basic Local Alignment Search Tool (BLAST) [20]. Paternal haplotypes were determined from the 44 male dogs in the group based upon a panel of six Y-STR markers, including *650.79.2*, *990.35.4*, *MS34A*, *MS34B*, *MS41A*, and *MS41B* [21, 22].

### Genomic STR markers and DLA class I and II STR markers

Thirty-three STR loci across 25 chromosomes were used to assess genomic diversity, while four STR loci were used to determine DLA class I haplotypes and three STR loci were used for DLA class II haplotypes. The primer sequences, dye markers, conditions for amplification and analysis of these STR markers have been published [23].

### The use of allele frequencies for standard genetic assessments

A genetic assessment using allele frequencies was conducted using GenAlEx 6.5 software [24]. The population statistics used in this study included Aa, Ae, Ho, He, and the inbreeding coefficient F. Aa represented the average number of alleles at each locus; Ae represented the average effective number of alleles at each locus; Ho is observed heterozygosity, while He is the expected heterozygosity if the population was randomly breeding. The value F is an inbreeding coefficient derived by [1-(Ho/He)]. An F value of 0 indicates that the population as a whole is in Hardy-Weinberg equilibrium (HWE), i.e., randomly breeding. A negative F value of −1.0 indicates that that every member of the population is genetically distinct, while a F value of +1.0 indicates that all members were genetically identical. Principal coordinate analysis (PCoA) was performed in Excel using the XLSTAT software.

Internal relatedness (IR) is a statistical estimate of how closely an individual dog’s parents were related to each other [23]. It also uses allele frequency data, but unlike Ho and He, it gives more weight to uncommon alleles. An IR value of −1.0 means both parents were totally unrelated, while a value of +1.0 means the parents were genetically identical. Internal relatedness values can also be used to plot the population as a whole and estimate the amount of genetic diversity lost as a result of breed creation [23]. The latter estimate is made by adjusting the frequencies of alleles found at each genomic STR locus to the frequency of the same allele in a large population of random breeding village dogs, thus yielding IR-village dog (IRVD). These village dogs breed randomly and have genetic links with most modern breeds, and they are one of the largest single reservoirs of ancestral diversity inherited by descent [22, 25, 26]. The IR and IRVD were graphed using the software R [27].

### Analysis of GWAS data for runs of homozygosity

Illumina 170 K CanineHD datasets for English bulldog and Standard poodle were obtained from other GWAS studies [28, 29] and filtered for minor allele frequency (<0.05) and genotype (>90 %). Ten English bulldogs and 10 Standard Poodles were randomly selected and their SNP arrays interrogated for runs of homozygosity (ROH) using PLINK [30]. Runs of homozygosity with allele sharing across all individuals were identified by applying the option *–homozyg-group*. The analysis yielded the number, size range of ROH, and the portion of ROH that was shared (consensus ROH) by all individuals interrogated. This information was extended by looking for the consensus ROH that were shared by 6–9 of the 10 individuals tested.

## Results

### Paternal haplotypes

Paternal haplotypes were determined for 44 male English Bulldogs in the study population using six Y-specific STRS. Haplotype 1 was dominant in 93.1 % of the dogs, while haplotype 2–4 were observed in 2.3 % of dogs each (Table 1). Paternal haplotypes three and four were closely related to the dominant haplotype 1 and appeared to arise from a single mutation in the MS41B STR locus, changing K to J or L, respectively. Test data from the UC Davis Veterinary Genetics Laboratory has also found the dominant haplotype 1 of the English bulldog in the French bulldog, Bull Terrier, Bull mastiff, Miniature bull terrier, Staffordshire bull terrier, Wire-haired fox terrier, Beagle, and Coton de Tulear.

**Table 1:** Paternal haplotypes detected in 44 male English bulldogs

Hap	#	Frequency	650.79.2	990.35.4	MS34A	MS34B	MS41A	MS41B
1	41	0.931	DK	E	J	G	C	K
2	1	0.023	DK	E	G	J	C	I
3	1	0.023	DK	E	J	G	C	J
4	1	0.023	DK	E	J	G	C	L

### Maternal haplotypes

Five maternal haplotypes were identified in the control population based on mitochondrial sequences. The sequences of the haplotypes observed in English bulldogs corresponded to GenBank Accession numbers as follows: *EBU-A* (GenBank:KP665923), *EBU-C* (GenBank:KP665928), *EBU-J* (GenBank:KP665924), *EBU-K* (GenBank:KP665914), and *EBU-7* (GenBank:KP665930). Maternal haplotype frequencies and variations of base pair positions are listed in Table 2. Three of the five haplotypes were found in 90.9 % of the dogs. The five maternal haplotypes identified in English bulldogs have been found among a number of common dog breeds, with the three most common matrilines (EBU-C, −J and –K) also found in mastiff- and brachycephalic-type breeds (Table 3).

**Table 2:** Frequencies and base pair position variations of maternal haplotypes found in English bulldogs

		Base pair position																
Hap	Frequency	124	125	133	140	145	152	156	163	165	166	223	313	328	425	468	516	538
EBU-A	0.045	T	C	T	A	T	G	G	T	A	A	C	C	C	T	T	/^a^	T
EBU-C	0.295	T	T	T	A	C	A	A	T	G	A	C	T	T	C	C	A	C
EBU-J	0.364	T	T	C	G	C	A	A	T	G	A	C	T	T	C	T	A	T
EBU-K	0.25	C	T	T	A	C	G	A	C	G	A	T	C	T	T	T	G	T
EBU-7	0.045	T	T	T	A	C	T	A	T	G	G	C	T	T	C	C	A	T

^a^The “/” indicates a base pair deletion mutation

**Table 3:** Breeds identified by the UC Davis Veterinary Genetics Laboratory that share maternal haplotypes with English bulldogs

Haplotype	Breeds exhibiting haplotype
EBU-A	Airedale Terrier, Australian Terrier, Basset Hound, Beagle, Bloodhound, Bolognese, Brittany Spaniel, Cardigan Welsh Corgi, Chihuahua, Coton de Tulear, English Bulldog, Golden Retriever, Great Pyrenees, Jack Russell Terrier, Labradoodle, Maltese, Poodle, Portuguese Water Dog, Shetland Sheepdog, Tibetan Spaniel, Yorkshire Terrier
EBU-C	Airedale Terrier, American Staffordshire Bull Terrier, Australian Shepherd, Brittany Spaniel, Bucovina Shepherd Dog, Chihuahua, Cocker Spaniel, Dachshund, Dwarf, Schnauzer, English Bulldog, English Springer Spaniel, Fox Terrier, Greyhound, Irish Setter, Labrador Retriever, Miniature Dachshund, Rottweiler, Saint Bernard, Shiba Inu, Siberian Husky
EBU-J	Airedale Terrier, American Pit Bull Terrier, Bichon Frise, Boxer, Brittany Spaniel, Bull Mastiff, Cavalier King Charles Spaniel, Dalmatian, English Bulldog, English Bull Terrier, German Shepherd, Greyhound, Jack Russell Terrier, Miniature Pinscher, Miniature Schnauzer, Pug
EBU-K	American Pit Bull Terrier, Australian Shepherd, Beagle, Black Russian Terrier, Blue Heeler, Border Collie, Boston Terrier, Brittany Spaniel, Cocker Spaniel, Collie, English bulldog, Havanese, Pomeranian, Shiba Inu, Shikoku, Swedish Elkhound, West Highland White Terrier
EBU-7	American Cocker Spaniel, Beagle, Bearded Collie, Briard, Cockapoo, Cocker Spaniel (unspecified), Dachshund, English Bulldog, Galgo, Jack Russell Terrier, Miniature Schnauzer, Shetland Sheepdog, Shiba Inu, Toy Poodle, Viszla, Welsh Springer Spaniel

### Genetic assessment of healthy and unhealthy English bulldogs using 33 genomic STR loci

A genetic assessment of 102 control English bulldogs based on the alleles and their frequencies at each of the 33 genomic STR loci was conducted and the population statistics were evaluated with GenAlex 6.51 software (Tables 4 and 5). The highest number of individual alleles found for a single autosomal STR locus was eleven (*VGL1165*), and the lowest was three (*INRA21*). Most of the loci had one or two alleles that dominated in frequency. Nineteen of 33 loci had single alleles with a frequency ≥ 50 %, which are highlighted in Table 4. Six of the STRs had one allele with a frequency of 70 % or greater, and allele 202 at locus *REN162C04* was virtually fixed with a frequency of 0.99 amongst the 102 dogs studied (Table 4). The average number of alleles per locus was 6.46, of which an average of 2.77 alleles per locus contributed disproportionately to overall diversity (Table 5). The observed and expected heterozygosity (Ho and He) were essentially the same, yielding an inbreeding coefficient (F) close to zero. The Ho, He and F values indicated that this population of 102 dogs was in HWE.

**Table 4:** Allele frequencies at 33 genomic STR loci for 102 control English bulldogs

VGL1165	AHT137	VGL1063	VGL0760	VGL0910	AHTh260	AHT121	VGL2409	VGL2918
17 (0.005)	133 (0.005)	12 (0.015)	14 (0.015)	13 (0.01)	242 (0.005)	94 (0.039)	14 (0.203)	13 (0.333)
19 (0.005)	135 (0.23)	13 (0.015)	15 (0.005)	17 (0.142)	244 (0.098)	96 (0.069)	15 (0.01)	14 (0.132)
21 (0.127)	137 (0.044)	14 (0.172)	20 (0.005)	18 (0.039)	246 (0.304)	98 (0.039)	16 (0.104)	15 (0.02)
22 (0.196)	139 (0.005)	15 (0.044)	21 (0.034)	19 (0.333)	248 (0.029)	100 (0.103)	17 (0.228)	17 (0.147)
23 (0.005)	141 (0.005)	16 (0.039)	22 (0.093)	20 (0.172)	250 (0.005)	**102 (0.676)**	18 (0.05)	18 (0.044)
25 (0.054)	143 (0.01)	17 (0.044)	23 (0.338)	21 (0.27)	252 (0.02)	104 (0.054)	19 (0.02)	19 (0.27)
26 (0.167)	**147 (0.569)**	18 (0.397)	24 (0.196)	22 (0.025)	254 (0.005)	106 (0.015)	20 (0.104)	20 (0.029)
27 (0.206)	149 (0.01)	19 (0.235)	25 (0.02)	23 (0.005)				
28 (0.206)	151 (0.078)	20 (0.034)	26 (0.005)					
29 (0.005)	153 (0.005)							
30 (0.005)								
REN105L03	AHTH130	FH2001	FH2054	REN169O18	VGL3008	INU055	AHTh171-A	
229 (0.01)	**119 (0.515)**	128 (0.005)	148 (0.01)	156 (0.127)	13 (0.49)	208 (0.005)	219 (0.441)	
231 (0.338)	121 (0.162)	132 (0.147)	152 (0.103)	160 (0.005)	14 (0.039)	210 (0.343)	223 (0.054)	
233 (0.083)	123 (0.005)	136 (0.005)	156 (0.113)	162 (0.078)	15 (0.103)	212 (0.025)	225 (0.431)	
235 (0.422)	125 (0.098)	140 (0.034)	160 (0.127)	164 (0.01)	18 (0.029)	214 (0.069)	227 (0.005)	
239 (0.044)	127 (0.088)	**144 (0.627)**	168 (0.279)	168 (0.005)	19 (0.279)	216 (0.147)	229 (0.025)	
241 (0.098)	129 (0.034)	148 (0.167)	172 (0.343)	**170 (0.77)**	20 (0.049)	218 (0.412)	237 (0.044)	
245 (0.005)	133 (0.098)	152 (0.015)	176 (0.025)	172 (0.005)	21 (0.01)			
VGL1828	VGL2009	VGL3235	AHTk253	C22.279	FH2848	INU005	REN54P11	
14 (0.152)	9 (0.005)	**13 (0.877)**	284 (0.005)	116 (0.098)	230 (0.162)	110 (0.025)	222 (0.025)	
15 (0.235)	10 (0.015)	14 (0.025)	286 (0.299)	**118 (0.564)**	**238 (0.539)**	122 (0.005)	226 (0.377)	
**16 (0.539)**	13 (0.049)	15 (0.01)	288 (0.441)	120 (0.108)	240 (0.098)	124 (0.211)	228 (0.01)	
17 (0.015)	**14 (0.809)**	16 (0.039)	290 (0.216)	124 (0.034)	242 (0.015)	**126 (0.578)**	232 (0.039)	
19 (0.049)	15 (0.113)	17 (0.025)	292 (0.039)	126 (0.196)	244 (0.186)	132 (0.181)	**236 (0.549)**	
20 (0.01)	16 (0.01)	18 (0.025)						
REN64E19	REN247M23	REN169D01	REN162C04	LEI004	INU030	AHTk211	INRA21	
143 (0.005)	266 (0.083)	202 (0.186)	200 (0.005)	83 (0.01)	144 (0.127)	**87 (0.76)**	95 (0.279)	
145 (0.392)	268 (0.24)	212 (0.225)	**202 (0.985)**	85 (0.064)	146 (0.005)	89 (0.015)	**97 (0.706)**	
147 (0.235)	270 (0.029)	**216 (0.564)**	206 (0.005)	**95 (0.757)**	148 (0.005)	91 (0.01)	101 (0.015)	
149 (0.005)	**272 (0.647)**	218 (0.025)	208 (0.005)	107 (0.168)	**150 (0.863)**	95 (0.216)		
153 (0.363)								

Bold data indicates allele frequencies over 50 %

**Table 5:** Average (Aa) and effective (Ae) alleles per locus of 33 autosomal STRs for case (*n* = 37) and control (*n* = 102) English bulldogs

Population		Aa	Ae	Ho	He	F
Case	Mean	5.182	2.770	0.575	0.570	−0.001
(*n* = 37)	SE	0.324	0.190	0.038	0.035	0.020
Control	Mean	6.455	2.772	0.572	0.574	0.006
(*n* = 102)	SE	0.385	0.198	0.033	0.032	0.012
Total	Mean	6.788	2.791	0.573	0.575	0.007
(*n* = 139)	SE	0.379	0.199	0.034	0.033	0.011

**Fig. 2:**
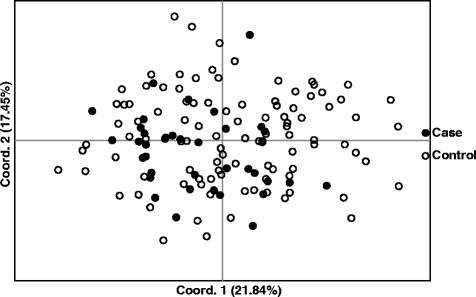
PCoA plot of 102 healthy English bulldogs (control) and 37 English bulldogs admitted to the VMTH for a variety of health problems (case)

Results from a genetic assessment of 37 unhealthy case dogs were compared to that of the 102 presumably healthy controls (Table 5). The two populations were essentially identical by all of the genetic parameters, with the exception of the average alleles per locus for case dogs, which was a lower in case dogs (Table 5). However, this difference was due to variation in sample size, because Aa was similar (5.182 vs 5.364) when the 37 case dogs were compared to 37 control dogs randomly selected from the larger population by Excel (data not shown). Observed and expected heterozygosity did not differ between case and control dogs and the values for F were near zero for both groups. No differences were detected between case and control populations by principal coordinate analysis (Fig. 2).

### Internal relatedness and adjusted internal relatedness

**Fig. 3:**
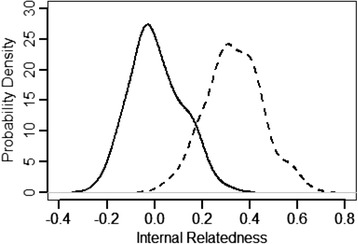
Internal relatedness (IR, *solid line*) and internal relatedness relative to village dogs (IRVD, *dash line*) of 102 English Bulldogs

The mean IR value for the 102 English Bulldogs was 0.007, with individuals ranging from−0.234 (most outbred) to +0.304 (most inbred) (Fig. 3, Table 6). This suggested that there were highly inbred individuals in the population, which were balanced by an equal portion of outbred dogs, giving the impression that the population as a whole was a product of random breeding.

**Table 6:** Summary of IR and IRVD values from English bulldogs (*n* = 102) and Standard Poodles (*n* = 664)

	IR		IRVD	
	EBU	ST	EBU	ST
Min.	−0.234	−0.306	0.043	−0.119
1st Qu	−0.063	−0.025	0.26	0.163
Median	−0.009	0.046	0.319	0.250
Mean	0.007	0.056	0.335	0.254
3rd Qu	0.085	0.126	0.41	0.328
Max.	0.304	0.528	0.64	0.732

The adjusted IR value (IR-village dog or IRVD) for the population gave a more accurate measure of just how inbred the parents of modern English bulldogs were compared to village- or landrace- type dogs from which the breed evolved. IRVD values ranged from 0.043 to 0.64 with a mean of 0.335 (Fig. 3, Table 6]. Therefore, 78/102 bulldogs (77 %) had IRVD values >0.25 and were more closely related to each other than offspring of full sibling parents from a random breeding village dog population. Values >0.25 would occur only if full sibling parents were offspring of inbred parents; the more inbred the parents the higher the IRVD score.

**Fig. 4:**
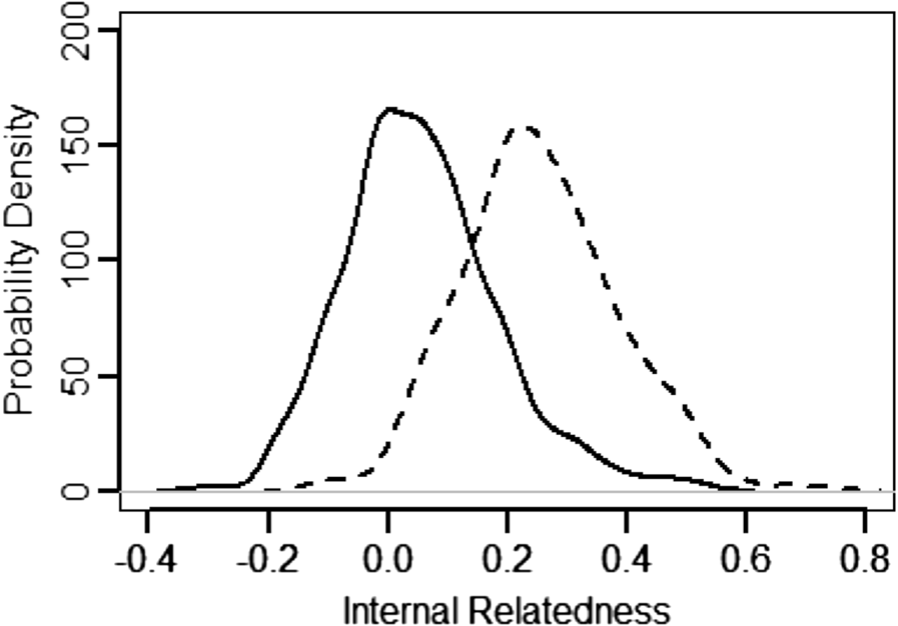
Internal relatedness (IR, *solid line*) and internal relatedness relative to village dogs (IRVD, *dash line*) of 664 American Standard Poodle

The IR and IRVD values for English bulldogs were further compared with the Standard Poodle (Fig. 4, Table 6). Standard Poodles like the English bulldog, are very popular and a sub-population has been extensively inbred for a uniform and desirable appearance [23]. A comparison of minimum and maximum IR values for the two breeds show parents of individual Standard Poodles to be both more unrelated (−0.306 vs−0.234) and related (0.304 vs 0.528) than parents of individual English bulldogs (Table 6). The differences are even more apparent when comparing IRVD values (Table 6). This comparison demonstrated that many Standard Poodles were offspring of parents that were even more inbred than parents of the most inbred English bulldogs. Although both breeds appear to be highly inbred, Standard Poodles have retained much more genetic diversity across the breed [23].

### Frequency of STR-associated DLA Class I and II haplotypes

Eleven DLA class I and nine class II haplotypes were identified among the 102 English bulldogs tested (Table 7). The dominant class I haplotype was observed in 40.7 % of the dogs, while the three most common haplotypes together accounted for nearly 80 % of all class I haplotypes. The dominant class II haplotype was found in 62.3 % of English bulldogs, while the two most common were present in 86 % of individuals. The three most common DLA class I and two most common class II haplotypes were homozygous in 24.5 and 50 % of dogs, respectively (Table 7).

**Table 7:** DLA class I and II haplotypes of 102 English bulldogs and their frequencies. The percentage homozygosity for each haplotype is also given

Class I			Class II		
Haplotype	Frequency	Homozygous	Haplotype	Frequency	Homozygous
388/369/289/188	0.407	18.63 %	343/324/282	0.623	42.16 %
382/371/277/183	0.216	3.92 %	343/322/280	0.235	7.84 %
388/369/289/186	0.172	1.96 %	339/322/284	0.088	0 %
382/371/277/178	0.108	0 %	345/327/288	0.025	0 %
386/383/289/186	0.044	0 %	339/327/282	0.01	0 %
380/371/277/186	0.025	0 %	339/322/280	0.005	0 %
375/373/287/180	0.01	0 %	343/324/268	0.005	0 %
376/369/291/178	0.005	0 %	343/325/286	0.005	0 %
376/379/277/181	0.005	0 %	351/327/280	0.005	0 %
386/369/277/186	0.005	0 %			
387/375/293/180	0.005	0 %			
Total homozygous		24.5 %			50 %

Individual alleles within the 7 STR loci that defined the DLA class I and II regions were assessed for heterozygosity. Each of seven loci was defined by one dominant and one semi-dominant allele and several minor alleles (Table 8). The most common alleles were usually found in the most prevalent DLA class I and II haplotypes.

**Table 8:** Allele frequencies for STR loci associated with DLA Class I and II regions in 102 English bulldogs

DLA Class I			
DLA I-3CCA	DLA I-4ACA	DLA I-4BCT	DLA1131
375 (0.01)	**369 (0.608)**	**277 (0.338)**	178 (0.093)
376 (0.01)	**371 (0.328)**	287 (0.01)	180 (0.015)
380 (0.025)	373 (0.01)	**289 (0.642)**	181 (0.005)
**382 (0.304)**	375 (0.005)	291 (0.005)	**183 (0.216)**
386 (0.049)	379 (0.005)	293 (0.005)	**186 (0.265)**
387 (0.005)	383 (0.044)		**188 (0.407)**
**388 (0.598)**			
DLA Class II			
5ACA	5ACT	5BCA	
339 (0.104)	**322 (0.332)**	268 (0.005)	
**343 (0.866)**	**324 (0.624)**	**280 (0.255)**	
345 (0.025)	325 (0.005)	**282 (0.623)**	
351 (0.005)	327 (0.04)	284 (0.088)	
		286 (0.005)	
		288 (0.025)	

Alleles occurring at the highest frequency at each locus were in bold

Although the DLA is in strong linkage disequilibrium compared to other regions of the genome, there are recombination hotspots within the DLA and fixation indices using allele frequencies at the seven class I and II STR loci is of some value (Table 9). The average DLA class I/II associated alleles per STR loci was 5.43, but only 2.16 (40 %) were contributing to most of the genetic diversity (Table 9). This was a reflection of the imbalance in the frequency and homozygosity of certain founding DLA class I and II haplotypes, although the observed and expected heterozygosity were similar and F was near zero. The neutral F value indicated that the imbalance in DLA classes I/II haplotypes was not a recent occurrence but resulted from small number of ancestral founders or artificial genetic bottlenecks much earlier in breed evolution. Periods of inbreeding associated with genetic bottlenecks such as popular sire effects are often followed by a return to random breeding, although the outcome may be a permanent reduction in founder size [23].

**Table 9:** Average (Aa) and effective (Ae) alleles per locus for 7 DLA class I and II STRs for 102 English bulldogs

	Aa	Ae	Ho	He	F
Mean	5.43	2.16	0.51	0.50	−0.02
SE	0.43	0.24	0.05	0.05	0.03

### Runs of homozygosity (ROH) in genomes of English bulldogs and Standard Poodles

The English bulldog is very different in outward appearance to breeds such as the Standard Poodle, which still resembles its Middle Eastern/SE Asian village dog and their European Landrace relatives in most aspects. Therefore, variations within genomes of the English bulldogs were compared with those of the Standard Poodle. The implication was that variations within the genome would be much greater in the English bulldog because the extreme conformational changes would require much more intense positive selection within specific parts of the genome. GWAS data from 10 randomly selected English bulldogs and Standard Poodles was obtained from previous studies [23, 29]. The level of inbreeding based on the proportion of SNPs that were homozygous in canine 170 K arrays was virtually identical in the two breeds; 0.65 ± 0.03 in English bulldogs and 0.63 ± 0.03 in Standard Poodles. The results of IR and IRVD comparisons also showed that highly inbred individuals existed in both breeds. Therefore, it can be assumed that variations in the genomes of English bulldogs and Standard Poodles were not associated with inbreeding per se, but to strong positive selection pressures in various regions of the genome related to comparative breed specific traits and to an associated loss of genetic diversity. The GWAS data was then used to estimate the number and size of ROH in the two breeds.

The largest consensus ROH was determined for all 10 dogs of each breed (Table 10). As expected, the number of large consensus ROH shared by 100 % of the population is low because different proportions of individuals will possess one or more heterozygous SNP that create variable sized runs of overlapping homozygosity within these larger ROH. The effect of this small amount of heterozygosity can be offset by identifying the largest consensus ROH shared by at least 9, 8, 7 or 6 out of 10 individuals in the population (Table 10).

**Table 10:** The total number of consensus runs of homozygosity (ROH) depending on proportion of dogs in the population among which they were shared, number of *Canis familiaris* autosomes (#CFA) on which ROH were identified and mean size and standard deviation in Mb of consensus ROH

	English bulldog				Standard poodle			
#dogs	#ROH	#Chr	Mean (Mb)	SD (Mb)	#ROH	#Chr	Mean (Mb)	SD (Mb)
10	2	2	3.68	1.46	0	0	0	0
9	8	7	1.79	1.36	1	1	0.11	0
8	16	12	2.02	1.97	3	3	1.96	1.83
7	39	20	2.08	2.32	5	2	0.93	1.41
6	53	28	1.31	1.18	21	16	1.33	0.83
Total	118	31	1.31–3.68		30	19	0.11–1.96	

Two consensus ROH 4.71 Mb and 2.65 Mb in size were identified on different chromosomes in 100 % of English bulldogs tested. The largest autosomal ROH in a single English bulldog was 58.24 Mb on CFA20. The largest consensus ROH on the X-chromosome (CFA39) was 51.35 Mb on five of the six male dogs in the group. The number of consensus ROH that were detected on autosomes increased as the number of dogs that shared larger consensus ROH decreased. Nine of ten of the dogs shared eight additional ROH compared to 10/10 dogs; 8/10 dogs an additional 16 ROH compared to 10/10 and 9/10 dogs; 7/10 dogs had 39 more ROH compared to 8/10, 9/10 and 10/10 dogs; and 6/10 dogs had 53 more ROH than 7/10, 8/10, 9/10 and 10/10 dogs (Table 10). In total, 118 consensus ROH ranging from 1.31 to 3.68 Mb on 31 different autosomes were shared by at least 6 of the 10 dogs studied (Table 10).

The number and size of ROH were much lower in Standard Poodles than English bulldogs (Table 10). No consensus ROH was shared among all 10 of the Standard Poodles and only one ROH of 0.11 Mb was shared by 9/10 of individuals. Twenty-one ROH on 16 chromosomes with consensus sizes of 1.33 ± 0.83 M were shared by at least 6/10 of the dogs. The largest ROH of a single Standard Poodle was 59.16 Mb on CFA11. The largest consensus ROH was 3.66 Mb on CFA30 shared by 8/10 Standard Poodles. A total of 30 consensus ROH on 19 chromosomes and 0.11–1.96 MB in size were shared by at least 6/10 Standard Poodles in the population tested. Therefore, ROH of English bulldogs were larger, more variable in size between individuals, more numerous, and involved many more chromosomes than those of Standard Poodles.

## Discussion

This study examined genetic diversity among contemporary English bulldogs used for breeding purposes from the USA (*n* = 87) and several other countries (*n* = 15). Thirty-seven pet English bulldogs seen for health problems at UC Davis Veterinary Clinical Services were also included in the study. The populations that were tested evolved from one major and three minor paternal haplotypes and three major and two minor maternal haplotypes. One paternal haplotype, which was found only in a one dog, appeared to be distinct. The two remaining minor paternal haplotypes, also in single dogs, differed from the dominant paternal haplotype by a simple mutation. This pattern of a single dominant paternal haplotype and a small number of maternal haplotypes has been observed in most pure breeds and is therefore not unique to English bulldogs [21, 25].

The paternal and maternal haplotypes identified in these English bulldogs provides a window into the breed’s ancestry. The dominant paternal haplotype occurs as expected in several bracycephalic breeds that include “bull” in their breed names, as well as breeds such as the Beagle, Coton de Tulear, and Wire-haired fox terrier. The minor unrelated haplotype has been found in a related genetic form in Akita and Beagles. The two minor mutant haplotypes have not been seen in any other breed suggesting that these have arisen by mutations within the breed. The major maternal haplotype EBU-J occurs in several of the brachycephalic breeds (Boxer, Pug, Boston Terrier) as well as small Mastiff-type dogs (Bull Mastiff, English Bull Terrier, American Pit Bull Terrier), while EBU-K has been found in Asian Mastiffs such as the Shiba Inu and Shikoku. Therefore, the maternal haplotypes seen in English bulldogs support the general belief that English bulldogs evolved from Mastiff-type dogs crossed with brachycephalic breeds such as the Pug [1]. It is tempting to associate paternal rather than maternal introgressions from smaller and much less aggressive non-brachycephalic breeds were used to make English bulldogs more acceptable as household pets.

Evidence obtained from the 33 genomic STR loci provides additional evidence for the small founder population and artificial genetic bottlenecks that led to the modern English bulldog. The average number of alleles per locus was similar to other pure breeds that have been diversity tested.^15^ However, an examination of the average effective alleles per locus indicates that only one or two alleles are actually contributing to the overall genotype and phenotype diversity of the breed. Six of 33 loci had an allele with frequency >70 %, and allele 202 at locus *REN162C04* was homozygous in 101/102 dogs studied. Loss of genetic diversity as a result of pure-breeding, especially when those breeds undergo selection for conformation, is well documented [25]. The various conformational changes used in creating breed-specific phenotypes often results in large regions of extended homozygosity across the genome [31–35]. The larger and more numerous these regions, the more often they will be associated with an STR and the more likely that certain STR alleles will be at higher frequency and also exist in a homozygous state.

Measurements of observed and expected heterozygosity (Ho and He) and the inbreeding coefficient F are also useful in looking at the genetic makeup of a population. Observed and expected heterozygosity were similar for the English bulldogs tested, yielding an inbreeding coefficient F that was close to zero (0.001). This indicated that the population as a whole was in HWE despite a limited gene pool and that English bulldog breeders were doing a reasonable job of identifying more distantly related dogs for mating. However, IR values indicated that He, Ho and F values were misleading, as many individual dogs in the study were actually products of parents that were much more related to each other than assumed from the population-wide fixation indices.

Internal relatedness has been widely used as an indicator of population fitness [36–42], implying that closely related parents reflect a loss of genetic diversity in the total population under study. The average IR value is 0.25 for a litter of puppies born to full sibling parents from a genetically diverse and randomly bred population. The average IR value for English bulldogs was around 0.007, however there were a number of individuals with IR values around 0.20. However, IR does not take into account the degree of genetic diversity that has been lost as a result of breed development. Virtually all of modern pure-breeds can trace their origins to village-type dogs that proliferated during the Neolithic era in the Middle East and SE Asia and populated other regions of the world [22, 26]. Allele and allele frequencies found in pure breeds can be adjusted to the frequencies of those same alleles found in village dog populations and used to re-calculate or adjust IR values to approximate the expected diversity if no founder effects or artificial bottlenecks occurred during a breed’s evolution. When IR values were adjusted using allele frequencies in village dogs, the mean IRVD value for English bulldogs rose to 0.34, with 50 % of dogs having even greater values. A mean value of 0.34 indicates that the average English bulldog is genetically equivalent to offspring of full sibling parents that came from a highly inbred subpopulation of village-type progenitors. This shift to the right of the IRVD compared to IR curve was also seen in Standard Poodles, but to a much less degree, reflecting the greater amount of initial or retained genetic diversity in Standard Poodles.

The low number of different DLA class I and II haplotypes in English bulldogs was associated with an imbalance in the relative frequency of each haplotype. Four of 11 class I and 3/9 class II haplotypes were found in over 90 % of the individual English bulldogs tested. Moreover, there was a high level of homozygosity among the dominant DLA class I (19 %) and II (42 %) haplotypes. Although not balanced in frequency, genetic assessment of the seven STR alleles associated with the DLA class I and II regions showed them to be randomly segregating at this time. This indicated that the over-representation of certain haplotypes occurred at the onset of breed creation as a result of small founder numbers, and/or that it was associated with artificial genetic bottlenecks that were subsequently masked by a return to random selection. Although the DLA region is only a small part of the genome, the importance of these haplotype imbalances, small haplotype numbers, and increased homozygosity should not be underestimated [23, 42]. The breed suffers greatly from allergies, immunodeficiency, and a number of autoimmune disorders, which may be a reflection of loss of balanced selection and heterozygote advantage in the DLA region.

In order to gauge the extent to which humans shaped the phenotype of English bulldogs from typical dogs, we decided to compare the English bulldog with a breed that was similarly inbred based on the number of homozygous SNPs identified by Illumina 170 K canine SNP arrays and IR scores, but outwardly similar in appearance to the ancestral dog. The Standard Poodle, which has been similarly studied [23], met the desired criteria. One hundred nineteen ROH with consensus sizes ranging from 1.31 to 3.68 Mb and shared by at least 6/10 dogs tested were identified on 30 of the 38 autosomes. This was compared to 31 ROH with consensus sizes ranging from 0.11 to 1.96 MB on 19 chromosomes for the Standard Poodle. Although a proportion of the ROH observed in both English bulldog and Standard Poodle can be attributed to natural selection pressures occurring over thousands of years [33, 34], the differences in ROH size and number are better explained by the comparative changes in outward appearance. The physical traits of English bulldogs, such as extreme brachycephaly, chondrodysplasia, skin furrowing, differences in tail structure, size and behavior, are extreme compared to the physical changes seen in Standard Poodles. These specific and extreme phenotypic traits required strong positive selection (strong sweeps) in specific regions of the genome [43], while ROH required for the Standard Poodle phenotype were not nearly as strong or widespread. The variation in ROH observed in the genome of English bulldogs and Standard Poodles were in line with the findings of others. Vaysse and colleagues [32] identified 44 genomic regions among 49 pure breeds that had undergone intense selection and 22 blocks of SNPs in certain breeds that extended over one million bases. Lindblad-Toh and a large group of investigators [31] compared the haplotype structure of the genome of the Boxer and 10 other breeds and found regions of linkage disequilibrium extending over several megabases within a breed and tens of kilobases between breeds.

We did not associate runs of homozygosity in English bulldogs with characteristic phenotypic traits but are confident based on previous studies that the greater genomic variation in English bulldogs compared to Standard Poodles reflected stronger human-directed selection in the former breed than in the latter. Pollinger and colleagues [44] also concluded that strong artificial selection for breed-defining traits have reduced variation within many regions of the genome. Associations between ROH and species/breed traits such as those found in English bulldogs have been demonstrated for human-directed selection in many pure breeds [29, 32, 33, 45, 46]. Brachycephaly is a prominent phenotypic trait in the English bulldog and not a naturally selected phenotype of ancestral village- or landrace-type dogs. A ROH around 500 kb on CFA1 was associated with brachycephaly in a study that compared a number of brachycephalic breeds [29]. A more recent study confirmed the existence of the brachycephaly-associated region on CFA1 in boxers, but also identified a >8 Mb ROH on CFA26 [44]. Variation in CFA10 has been linked to ear morphology and body mass in a number of breeds [45]. Genomic regions of positive selection in dog breeds have also been associated with adaption to a diet richer in starch [35]. Vaysse and colleagues [32] also identified runs of homozygosity associated with breed-defining characteristics such as chondrodysplasia in Dachshund [590 Kb] and wrinkled skin in Sharpei [1.4 Mb], characteristics of English bulldogs but not Standard Poodles. They also concluded that artificial selection in domestic animals targeted different functional categories than natural selection. Pollinger et al., [44] identified a 40 Mb selective sweep on CFA11 associated with black coat color in Large Munsterlander and a 10 MB region on CFA3 in Dachshund containing *FGFR3*, which is responsible for achondroplasia in humans and presumably linked with related genes responsible for canine chondrodysplasia.

It can be assumed from this and other studies that the small founder population of the English bulldog, estimated at 68 individuals,^16^ coupled with human created artificial genetic bottlenecks have greatly diminished genetic diversity and fostered a wide range of health problems. Small founder numbers and artificial bottlenecks are a much more powerful cause of lost genetic diversity than inbreeding [33]. Some bulldog breeders from the UK have already realized that the artificial selection process had gone too far, either on their own or bowed by public pressure, and have revised breed standards that discourage physical features “that might prevent a dog breathing, walking and seeing freely” [46]. However, the brachycephalic syndrome in English bulldogs is much more complex than a “shortened head.” Breeds such as the Boxer are similarly brachycephalic but do not suffer to the same degree. In the case of the English bulldog, the nostrils are narrow, the base of the tongue is large and broad, the palate elongated and thickened, thus allowing for blockage of the pharynx especially during sleep [8–11]. The tracheas are usually hypoplastic (narrow), further compromising normal ventilation [47]. The constant pressures on the upper airways created by these obstructions may also damage and weaken the laryngeal muscles, cause eversion of the lateral ventricles, and further decrease pharyngeal patency [48]. The laryngeal collapse is in turn associated with bronchial collapse [49]. Therefore, the breathing problems in the English bulldogs go beyond mere shortening of the face and require specific changes in the nares, rostral skull, tongue, oropharynx and trachea. Concentrating on the brachycephalic syndrome also ignores other serious problems in the breed, such as inability to breed and deliver normally, poor mothering, high puppy mortality, the accumulation of simple recessive deleterious traits, a number of orthopedic problems, certain cancers, allergies, immunodeficiency, and autoimmune disorders. The authors would agree with O’Neill and colleagues [50] that breeding reforms should target commonly-diagnosed complex disorders that are amenable to genetic improvement and should place special focus on at-risk breeds. Unfortunately, in the case of English bulldogs, this list is very long, but it may still be possible to target the most serious of these disorders for genetic correction.

Assuming that there is a will to improve the overall health of English bulldogs, the question raised by this study is whether or not there is sufficient genotypic diversity remaining in the breed to allow “reverse genetics” to correct phenotypic abnormalities that have major impacts on health. There are certainly phenotypic differences that still exist between various lines of English bulldogs and among individuals, and many English bulldogs enjoy much better health than others. The regions flanking consensus ROH vary greatly in size in individual English bulldogs, suggesting that some of these regions may contain “hidden” genetic diversity that may prove critical for reversing the degree of brachycephaly or chondrodysplasia. The existence of phenotypic variation within the breed is evident from photographs of modern English bulldogs.^7,8^ The one region of the genome of English bulldogs that is least amenable to reverse genetics may be the DLA, which has very low diversity based on DLA class I and II haplotypes and a high level of homozygosity. The nine DLA class II haplotypes recognized in this group of English bull dogs were only a fraction of the 88 haplotypes that were reported for dogs in 2007 [51]. Genes within the DLA are important in regulating self/non-self recognition and immune responses and play a role in autoimmune disorders, allergies, and immun.

Populations that have lost genetic diversity through small founder numbers and artificial genetic bottlenecks are more likely to accumulate deleterious traits [33, 52]. A lack of genetic diversity also makes it harder to eliminate deleterious traits from a population once they are recognized. The mutation responsible for hyperuricosuria is carried by 25.5 % of English bulldogs and 3.1 % of the breed are homozygous and excrete uric acid [53]. Elimination of this recessive mutation from the breed could lead to a significant loss of breed-wide genetic diversity.

A low level of breed-wide genetic diversity also limits the ability to rapidly introduce desired traits, usually in the form of simple recessive mutations. There is increased demand for smaller and more compact English bulldogs, dogs with wrinkled coats and rare coat colors. Such refinements in the breed create popular sire effects and yet more artificial genetic bottlenecks that will cause the loss of more genetic diversity if not properly managed. The negative effects of the rapid introduction of new genetic traits on the health have been best documented in the “The Rare Color Bulldog Craze”.^17^ Indeed, English bulldog breeders appear to be more interested in adding recessive coat color mutations to increase puppy value than eliminating known deleterious mutations. English bulldog breeders around the world ordered 2482 tests from VGL UC Davis involving coat color between 2012 and 2016 compared to 62 tests for the hyperuricosuria mutation.^18^

In conclusion, English bulldog breeders differ widely on their perception of health problems in their breed and what do about them. Some breeders blame disreputable or backyard “commercial” breeders for the unhealthy dogs that are being sold.^19^^,^^20^ However, genetic differences were not observed between pet English bulldogs seen at the UC Davis Veterinary Clinical Services and presumably healthy breeding dogs being genetically tested for certain traits. Healthy and unhealthy bulldogs shared the same alleles at genomic STR loci and the allele frequencies are virtually identical. The same was true for DLA class I and II haplotypes. Other English bulldog breeders believe that the health of the breed can be improved by breeding from within existing bloodlines, although there has been little movement by breeders to embrace this concept. This is fueled by purists that vigorously argue that any deviation from the original standards is no longer a British (English) bulldog [47], even though the breed has continued to evolve in appearance over centuries and even the last few decades. Still others believe that health cannot be restored from within the breed without resorting to outcrossing.^12,13^

The feelings of individual English bulldog breeders about the health of their breed and what if anything should be done about it may ultimately be taken out of their hands. English bulldog breeders across the world must take seriously constitutional amendments on the rights of animals. The European Union has recently updated their rules on animal welfare in 2015.^21^ Although it was written specifically for farm animals; it holds that “animals” have rights of ‘freedom from discomfort” and ‘freedom from pain, injury and disease.” The EU rules on animal welfare have been restated in much greater detail by a 2013 constitutional amendment in Switzerland, which extended such rights to all animals [54]. Although it has not been uniformly enforced, many Swiss breeders have proactively begun outcrossing English bulldogs with the Olde English Bulldogge to create what is known as the “Continental Bulldog”,^22^ which will help bring the breed into compliance.

## Conclusions

Breeding of the English bulldog for extremes of brachycephaly, chondrodysplasia, skin folding and child-like appearance and personality has required a level of human-directed positive selection that has made the English bulldog both one of the most popular and unhealthiest of dog breeds. A DNA-based assessment of the breed along a number of parameters has confirmed that the breed is greatly lacking in genetic diversity, which may preclude or minimize the ability of breeders to recreate healthier phenotypes from existing genetic stock, to eliminate deleterious mutations, and to add in new phenotypic traits.

## Abbreviations

AKC, American Kennel Club; DLA, dog Leukocyte antigen; IR, internal relatedness; STR, short tandem repeat; VGL, veterinary genetics laboratory, UC Davis

## Footnotes

1 Breed Information Centre. Bulldog. http://www.thekennelclub.org.uk/services/public/breed/display.aspx?id=4084, accessed 08/02/2016.

2 The American Kennel Club: Get To Know The Bulldog. http://www.akc.org/dog-breeds/bulldog/, accessed on 26/06/2016.

3 Olde English Bulldogge, https://en.wikipedia.org/wiki/Olde_English_Bulldogge, accessed 26/06/2016.

4 Images of English Bulldogs through history, https://www.google.com/search?q=images+of+english+bulldogs+through+history&biw=1539&bih=703&tbm=isch&tbo=u&source=univ&sa=X&ved=0ahUKEwiX28n6-YTNAhVGLmMKHfGSAuQQsAQIGw, accessed 26/06/2016.

5 English bulldogs have been screwed for a long time. http://retrieverman.net/2012/04/26/english-bulldogs-have-been-screwed-for-a-long-time/, accessed 26/06/2016.

6 Images for English bulldogs from 19th century. https://www.google.com/#q=pictures+of+English+bulldogs+from+19th+century, accessed 26/06/2016.

7 Images of English bulldogs. https://www.google.com/search?q=images+of+english+bulldogs&biw=1559&bih=744&tbm=isch&tbo=u&source=univ&sa=X&sqi=2&ved=0ahUKEwiMz_KorunKAhVM6GMKHVdOD9IQsAQIGw&dpr=1, accessed 26/06/2016.

8 https://www.google.com/search?q=english+bulldogs+in+switzerland&biw=1920&bih=940&site=webhp&tbm=isch&tbo=u&source=univ&sa=X&ved=0ahUKEwjEkf6PzcjMAhUP42MKHR2QAYgQsAQINw, accessed 26/06/2016.

9 Why are bulldog puppies so expensive? braselsbulldogs.tripod.com/id38.htm, accessed 08/02/2016.

10 Dog Breed Info Center. Water (Walrus) Puppies (Anasarca puppies). www.dogbreedinfo.com/whelpingwaterpuppies.htm, accessed 26/06/2016.

11 Report from the Kennel Club/British Small Animal Veterinary Association Scientific Committee. Summary results of the purebred dog health survey for British bulldogs. http://www.thekennelclub.org.uk/media/16342/british%20bulldog.pdf, accessed 26/06/2016.

12 Britain’s Kennel Club announces sweeping reforms. http://latimesblogs.latimes.com/unleashed/2009/01/britains-kennel.html, accessed 27/06/2016.

13 A petition from the campaign to Save the English Bulldog Breed. Sign the Petition to: Department of Agriculture The Netherlands, Secretary of State: Dijksma. https://www.causes.com/actions/1742598-save-the-english-bulldog-breed, accessed 27/06/2016.

14 Airlines Ban Bulldogs, Snub-Nosed Breeds From Flying Because Of Health Risks To Dogs. http://www.huffingtonpost.com/2011/10/10/bulldogs-banned-airlines-health-risks_n_1003495.html, accessed 27/06/2016.

15 Canine genetic diversity. https://www.vgl.ucdavis.edu/services/dog.php, accessed 27/06/2016.

16 Bulldog (English bulldog). http://www.dogbreedhealth.com/bulldog/, accessed 27/06/2016.

17 The Rare Color BullDog Craze.https://www.musclebully.com/general/the-rare-color-bulldog-craze/, accessed 27/06/2016.

18 Veterinary Genetics Laboratory, UC Davis. The records of this laboratory show that English bulldog owners ordered 2482 tests involving coat color between 2012 and 2016 compared to 62 tests involving the hyperuricosuria mutation.

19 Why purchase a bulldog puppy from a reputable breeder? http://www.bulldogsworld.com/puppy/why-purchase-bulldog-puppy-reputable-breeder, accessed 27/06/2016.

20 How to breed English bulldogs. http://www.wikihow.com/Breed-English-Bulldogs, accessed 27/06/2016.

21 Animal welfare. http://ec.europa.eu/food/animals/welfare/index_en.htm, accessed 27/06/2016.

22 Continental bulldog. https://en.wikipedia.org/wiki/Continental_bulldog, accessed 27/06/2016.
